# Acid Sphingomyelinase Impacts Canonical Transient Receptor Potential Channels 6 (TRPC6) Activity in Primary Neuronal Systems

**DOI:** 10.3390/cells9112502

**Published:** 2020-11-18

**Authors:** Stefanie Zeitler, Fabian Schumacher, Juliana Monti, Daniela Anni, Debarpan Guhathakurta, Burkhard Kleuser, Kristina Friedland, Anna Fejtová, Johannes Kornhuber, Cosima Rhein

**Affiliations:** 1 Department of Psychiatry and Psychotherapy, Friedrich-Alexander-Universität Erlangen-Nürnberg (FAU), 91054 Erlangen, Germany; Stefanie.Zeitler@web.de (S.Z.); Juliana.Monti@uk-erlangen.de (J.M.); Daniela.Anni@hotmail.com (D.A.); Debarpan.Guhathakurta@uk-erlangen.de (D.G.); Anna.Fejtova@uk-erlangen.de (A.F.); johannes.kornhuber@uk-erlangen.de (J.K.); 2 Department of Toxicology, University of Potsdam, 14558 Nuthetal, Germany; fabian.schumacher@fu-berlin.de; 3 Department of Pharmacology & Toxicology, Institute of Pharmacy, Freie Universität Berlin, 14195 Berlin, Germany; kleuser@zedat.fu-berlin.de; 4 Institute of Molecular Biology, University of Duisburg-Essen, 45147 Essen, Germany; 5 Institute for Pharmacy and Biochemistry, Johannes-Gutenberg Universität Mainz, 55128 Mainz, Germany; kfriedla@uni-mainz.de; 6 Department of Psychosomatic Medicine and Psychotherapy, Friedrich-Alexander-Universität Erlangen-Nürnberg (FAU), 91054 Erlangen, Germany

**Keywords:** acid sphingomyelinase, sphingolipids, trpc6, major depressive disorder

## Abstract

The acid sphingomyelinase (ASM)/ceramide system exhibits a crucial role in the pathology of major depressive disorder (MDD). ASM hydrolyzes the abundant membrane lipid sphingomyelin to ceramide that regulates the clustering of membrane proteins via microdomain and lipid raft organization. Several commonly used antidepressants, such as fluoxetine, rely on the functional inhibition of ASM in terms of their antidepressive pharmacological effects. Transient receptor potential canonical 6 (TRPC6) ion channels are located in the plasma membrane of neurons and serve as receptors for hyperforin, a phytochemical constituent of the antidepressive herbal remedy St. John’s wort. TRPC6 channels are involved in the regulation of neuronal plasticity, which likely contributes to their antidepressant effect. In this work, we investigated the impact of reduced ASM activity on the TRPC6 function in neurons. A lipidomic analysis of cortical brain tissue of ASM deficient mice revealed a decrease in ceramide/sphingomyelin molar ratio and an increase in sphingosine. In neurons with ASM deletion, hyperforin-mediated Ca^2+^-influx via TRPC6 was decreased. Consequently, downstream activation of nuclear phospho-cAMP response element-binding protein (pCREB) was changed, a transcriptional factor involved in neuronal plasticity. Our study underlines the importance of balanced ASM activity, as well as sphingolipidome composition for optimal TRPC6 function. A better understanding of the interaction of the ASM/ceramide and TRPC6 systems could help to draw conclusions about the pathology of MDD.

## 1. Introduction

Recent studies link acid sphingomyelinase (ASM) to the pathology of major depressive disorder (MDD) [1,2,3]. The enzyme ASM (EC 3.1.4.12, sphingomyelin phosphodiesterase 1, *Smpd1*, optimum pH 5.0) cleaves sphingomyelin to ceramide [4], which regulates cell signaling, and besides other sphingolipids, is a crucial component of cell membranes [5,6,7]. Therefore, ASM is necessary for the equilibrium of a healthy sphingolipidome, and consequently, cell membrane constitution. Ceramide is the central lipid of the whole sphingolipid system, which includes many enzymes and multiple molecules [8]. It can be generated via various balanced pathways, as the N-acylation of sphingosine (as part of the ceramide *de novo* synthesis), the salvage pathway, and the degradation pathways of sphingomyelin and complex sphingolipids [9]. ASM is part of the degradation pathway of sphingolipids to form ceramide. Besides ASM, another sphingomyelinase can be found in neurons and is catalytically active at neutral pH (nSMase2) [10,11]. Nevertheless, until now, little has been known about the involvement of nSMase2 in MDD pathology. Lysosomal ASM (L-ASM) is present at the inner leaflet of the lysosome, as well as at the plasma membrane after translocation, due to apoptotic CD95 activation or specific stress stimuli [10,12,13]. Furthermore, ASM has been found residing in extracellular particles, thus also impacting on distant cells [14]. Due to its glycosylation pattern, secretory ASM (S-ASM) can also be secreted into the extracellular space [15,16].

In 2005, Kornhuber and colleagues revealed that ASM activity in peripheral blood mononuclear cells was elevated in depressed patients. When they cultured blood mononuclear cells of healthy volunteers and treated them with the antidepressive drugs imipramine and amitriptyline, ASM activity was significantly reduced [2]. In the following years, it was shown that many commonly used antidepressants, as well as further drug classes, functionally inhibit ASM activity (functional inhibitors of ASM activity: FIASMAs) [17,18]. FIASMAs promote the degradation of the ASM in the lysosomal compartment via inhibition of the electrostatic binding of the enzyme to the inner lysosomal membrane [19]. Recently, a mouse study confirmed that the antidepressive action of commonly used drugs for the treatment of MDD (as amitriptyline or fluoxetine) depended on ASM inhibition [1]. In a model of stress-induced depression using wildtype mice, FIASMAs decreased the levels of ceramide in the hippocampus. Furthermore, this treatment also increased neurogenesis, neuronal maturation, neuronal survival, and beneficially affected the behavior of these animals. Vice-versa, neuronal proliferation, maturation, survival was reduced in the presence of an artificial abundance of ceramide in murine hippocampi, due to the direct injection of ceramide 16:0, inhibition of ceramide metabolism using a blocker, or genetic modifications of animals. Furthermore, animals that genetically overexpressed ASM showed depression-like behavior already without any stress-induction, which could be rescued by the application of FIASMAs. In turn, animals fully lacking the ASM gene (ASM KO) had lower ceramide concentrations and lacked the described FIASMA-mediated effects. These animals exhibited less depression-like behavior than wildtype mice, illustrating that an ASM balance could be crucial for healthy brain physiology, especially in the context of MDD [1].

The proposed model of lipid raft microdomains might serve as an approach to explain how changes in ASM activity could impact MDD pathology mechanistically. Membrane lipids as sphingolipids and cholesterol confluence to rafts or microdomains, and bind membrane molecules, thus impacting membrane organization, which modifies kinetics, membrane localization, and/or trafficking of ion channels and receptors [20]. Due to its influence on the sphingolipidome composition, ASM has a great impact on some plasma membrane located molecules [12].

Members of the canonical transient receptor potential family (TRPC) are of high importance for controlling cellular functions and the support of depolarization to regulate neuronal activity in the brain by induction of a cation flux [21]. TRPC channels have been widely reviewed (see, for example, Reference [21]). TRPC4 and TRPC5, for instance, are important for a negative control of neuronal plasticity, especially during brain development [21]. Furthermore, inhibition of TRPC4 and/or TRPC5 could be an interesting target for the treatment of anxiety disorder [22]. TRPC3, TRPC6, and TRPC7 belong to the DAG-sensitive group, and therefore, are involved in many neuronal functions in response to neuronal receptor stimulation as G*_q_*_/11_ type G-protein-coupled receptors or receptor tyrosine kinases, thereby beneficially affecting neuronal and synaptic plasticity [21]. Additionally, they also play important roles in several neuronal diseases and psychiatric disorders [21]. In particular, the membrane-associated cation channel canonical transient receptor potential 6 (TRPC6) is crucially involved in neuroprotection, neuronal outgrowth, and synaptic plasticity [23,24,25,26,27,28,29], as well as in antidepressive action of hyperforin, the antidepressant active constituent of herbal antidepressant St. John’s wort [30]. Sphingomyelinases and ceramide were shown to control TRPC6 subcellular localization in lung endothelial and arterial smooth muscle cells, likely by their recruitment to specific plasma membrane domains [31,32].

Recently, we have demonstrated an interaction between ASM activity and TRPC6 function in rat pheochromocytoma 12 (PC12) cells. However, the link between TRPC6 and ASM remained correlative [33]. Therefore, in this study, we investigated the function of TRPC6 channels in neurons derived from ASM deficient mice (ASM KO) [34]. Our findings extend previous results found in PC12 cells. We propose that in primary neuronal cells, a physiological sphingolipidome balance is crucial for healthy functions of neuronal membrane proteins as TRPC6.

## 2. Materials and Methods

### 2.1. Rat Neuronal Cell Culture

Menzel glass cover slips (Ø 18 mm; VWR International #631–1342; Radnor, PA, USA) were coated with poly-L-lysine solution [0.5 mg/mL poly-L-lysine (Sigma #P1524; Sigma-Aldrich/Merck, Darmstadt, Germany) in 150 mM borate buffer pH 8.4] and washed with MQ. After coating, cover slips were placed in 12-well plates (TPP #92012; Techno Plastic Products AG, Trasadingen, Switzerland). Embryos of albino Sprague Dawley rats (RjHan:SD; Janvier Labs, Le Genest-Saint-Isle, France) were collected at E 18; rat neuronal cells were isolated and cultured as earlier described [35]. In brief, embryonic rat brains were collected, cortices were separated and placed in cold HBSS-/- (GIBCO #14175-053; Thermo Fisher Scientific, Waltham, MA, USA). Cortices were washed in HBSS-/- and tissue was incubated at 37 °C for 20 min in trypsin 0.25% (*w*/*v*) (GIBCO #15090-046). Afterward, cells were washed with HBSS-/-, 0.1 mg/mL DNase I (Roche #11284932001, Hoffmann-La Roche AG, Basel, Switzerland) was added and cells were triturated with a yellow cannula (Ø 0.9 × 40 mm 20 Gx1½” Gr.1, Braun #C721.1; via Carl-Roth, Karlsruhe, Germany) and with a brown cannula (Ø 0.45 × 25 mm 26 Gx1” Gr.18, Braun #C718.1). The cell suspension was filtered through a 100 µm nylon cell strainer (Corning Incorporation #352360; Corning Incorporation, New York, NY, USA), and healthy neurons were counted in a Neubauer counting chamber with trypan blue. Cells were suspended in DMEM-media [DMEM (GIBCO #41966029), 10% (*v*/*v*) FCS (Biochrom GmbH #S00115; Berlin, Germany), 1% (*v*/*v*) L-Glutamine (GIBCO #25030-024), 1% (*v*/*v*) anti/anti (GIBCO #15240-062)] to gain 100,000 cells/mL and plated. The DMEM-media was replaced 24 h past plating with NB-media mix [Neurobasal (GIBCO #12348-017), 0.4% (*v*/*v*) L-Glutamine, 2% (*v*/*v*) B27 50 x (GIBCO #17504-044), 1% (*v*/*v*) anti/anti]. Cortical neurons were used for experiments at DIV 20-23.

### 2.2. ASM Mouse Strain and Murine Cell Culture

Mice were bred with a heterozygosity for ASM deficiency [34], and a C57BL/6J background. For experiments, full ASM-knockout (ASM KO) and ASM-wildtype (ASM WT) siblings were used.

For mouse cell culture, a protocol was established based on earlier methods [36]. Newborn pups between P 0–P 1 were sacrificed by cephalic-segregation, cortices were taken immediately and placed into ice-cold HBSS-/-. HBSS-/-was discarded completely, and tissue was mixed with PPD-mix [HBSS-/-, 0.01% papain (*w*/*v*) (Worthington #LK003176; Worthington Biochemical Corporation, Lakewood, NJ, USA), 0.1% (*w*/*v*) dispase II (Roche #04942078001), 0.01% (*w*/*v*) DNase I (Worthington #LS0002139), 12.4 mM MgSO_4_] [37]. Cells were placed in a water bath at 37 °C. 10 min later, tissue was triturated. This trituration procedure was repeated twice. After the third trituration, cells were filtered through a 70 μm nylon cell strainer (Corning #352350) and the filter was washed once with 1 mL of NBA-media mix [Neurobasal A (GIBCO #12349-015), 1% (*v*/*v*) GlutaMax (GIBCO #35050-038), 2% (*v*/*v*) B27 50x, 1% (*v*/*v*) sodium pyruvate (GIBCO #11360-070), 1% (*v*/*v*) anti/anti]. The cell suspension was then centrifuged 5 min at 120× *g*. The supernatant was discarded, and the cell pellet triturated with 1 mL of NBA-media mix. This washing step was repeated twice. Cells were counted with a Neubauer counting chamber, and 200,000 cells were plated onto cover slips coated with poly-L-lysine [2.5 mg/mL poly-L-lysine in 150 mM borate buffer pH 8.4], which had been placed in 12-well plates. Cells were incubated at 37 °C, 5% CO_2_, humidified atmosphere, and used for experiments at DIV 13.

A modified protocol [38] of the original Banker’s culture [39] was applied. Neurons, derived from ASM KO or ASM WT mice, were cultured concomitantly with ASM WT glial cells.

For the preparation of glial cells, newborn pups were used between P 0 to P 3. Pups were sacrificed by cephalic segregation, and forebrains were taken immediately in cold HBSS-/-. Forebrains were washed once with cold HBSS-/-, trypsinated to create a 0.25% (*w*/*v*) trypsin solution, and incubated for 15 min at 37 °C. Cells were washed with HBSS-/-, subsequently 0.1 mg/mL DNase I was added. Tissue was triturated with a yellow and a brown cannula. 1 mL cell suspension was added together with 9 mL DMEM-media and filled into a T 75 flask (TPP #90076). Cells were incubated at 37 °C, 5% CO_2_, in a humidified atmosphere. The media was changed one day and every three to four following days after plating. As soon as the cells became confluent, they were split 1:2. After another seven days of culturing, cells were harvested and frozen for long-term storage.

Glial cells were thawed and plated into a 6 cm petri dish (TPP #93060) with plating media six to seven days prior the start of the next experiment [DMEM (GIBCO #41966029), 10% (*v*/*v*) FCS (Biochrom GmbH #S00115; Berlin, Germany), 0.5% (*v*/*v*) L-Glutamine (GIBCO #25030-024), 1% (*v*/*v*) anti/anti] and incubated at 37 °C, 5% CO_2_, humidified atmosphere.

For the preparation of neurons, newborn pups were used between P 0 to P 1. The pups were sacrificed by cephalic-segregation, and cortices were taken immediately in cold HBSS-/-. Tissue was washed once with cold HBSS-/- and trypsin was added to incubate for 15 min at 37 °C. The trypsin solution was removed, and cells were washed with HBSS-/-. Plating media was added together with DNase I 0.1 mg/mL to cortices, and tissue was dissociated by the use of a yellow and a brown cannula. Cells were rinsed through a 70 μm nylon filter. Neurons were counted using a Neubauer counting chamber and diluted to obtain 100,000 cells/cover slip. Cells were plated onto PLL-coated menzel glass cover slips, which were also equipped with three small paraffin dots at a 120° angle and incubated at 37 °C, 5% CO_2_, humidified atmosphere.

The media of the glial cells in the 6 cm petri dishes was changed to Banker’s-NBA-media mix (Neurobasal A, 2% (*v*/*v*) GlutaMax, 2% (*v*/*v*) B27 50x, 1% (*v*/*v*) sodium pyruvate, 1% (*v*/*v*) anti/anti). After 1 h incubation of cortical neurons on cover slips, cover slips were transferred upside down to lie on the surface of glial cells in the 6 cm petri dishes and finally incubated at 37 °C, 5% CO_2_ in a humidified atmosphere. On day one and day three after plating, 2 μL Ara-C (stock conc. 1.5 mM in MQ, Sigma #C1768) was added to the Banker’s culture to avoid further growth of glial cells. Neurons were used for experiments at DIV 12.

### 2.3. Genotyping of Mice

Tail cuts were collected, and KAPA HotStart Mouse Genotyping Kit (Roche #KK7352, kit includes: 10x KAPA Express Extract Buffer, 1 U/μL KAPA Express Extract Enzyme, 2X KAPA2G Fast HotStart Genotyping) was used for genotyping.

Tissue was mixed with 88 μL sterile MQ, 10 μL of 10x KAPA Express Extract Buffer, 2 μL of 1 U/μL KAPA Express Extract Enzyme. Samples were placed in a ThermoCycler (SensoQuest GmbH; Göttingen, Germany) (15 min at 75 °C) for lysis, while afterward, enzymes were inactivated at 95 °C for 5 min. For the PCR reaction, 1 μL of DNA extract, 12.5 μL 2x KAPA2G FAST HotStart Genotyping Mix, 1 μL of 10 pmol/μL Primer #ASM-PS 5′ (*AGCCGTGTCCTCTTCCTTAC*; Eurofins Scientific, Luxembourg, Luxembourg), 0.5 μL of 10 pmol/μL Primer #ASM PA1 3′ (*CGAGACTGTTGCCAGACATC*; Eurofins Scientific), 0.5 μL of 10 pmol/μL Primer #ASM PA2 3′ (*GGCTACCCGTGATATTGCTG*; Eurofins Scientific), and 9.5 μL MQ were mixed. PCR settings were: 3 min at 95 °C, 35 cycles of 15 s at 95 °C/15 s at 58 °C/30 s at 72 °C, 3 min at 72 °C, and a holding temperature of 15 °C in a ThermoCycler. A 1.5% (*w*/*v*) agarose gel (PeqLab Co. #351.020; Shanghai, China) was prepared containing ethidium bromide (Carl-Roth #HP47.1). The gel was loaded with samples and run at 120 V for 35 min. Staining was analyzed using a gel documentation device with UV (Gel Stick Touch device; INTAS, Göttingen, Germany). A band at 523 bp represented the genotype “ASM KO”, and a lower band at 269 bp “ASM WT”.

### 2.4. Enzymatic Activity Assay

Enzymatic activity of ASM in murine tissue was investigated as earlier described [40].

### 2.5. Lipid Quantification by Liquid Chromatography Tandem-Mass Spectrometry (LC-MS/MS)

Samples of murine frontal cortices were homogenized in an aqueous buffered solution using a Bead Ruptor 12 (Omni International, Kennesaw, GA, USA). Aliquots of the homogenates, which corresponded to tissue equivalents of 1 mg for analysis of sphingolipids or 2 mg for determination of diacylglycerols (DAG), were subjected to lipid extraction using methanol/chloroform (2:1, *v*/*v*) as described previously [41]. The extraction solvent contained either sphingosine-d_7_ (Sph-d_7_), sphingosine 1-phosphate-d_7_ (S1P-d_7_), ceramide d18:1/17:0 (C17 Cer) and sphingomyelin d18:1/16:0-d_31_ (C16-d_31_ SM), or 1,3-dipentadecanoin (15:0/15:0 DAG) and 1,3-diheptadecanoin-d_5_ (17:0/17:0-d_5_ DAG) as internal standards. After lipid extraction, samples were saponified using methanolic potassium hydroxide and later neutralized with glacial acetic acid prior to sample concentration. For DAG analysis, saponification and neutralization were omitted. Lipids were chromatographically separated with an Agilent 1260 Infinity HPLC system (Agilent Technologies, Waldbronn, Germany) coupled to either an Agilent 6490 triple-quadrupole MS (for Sph and S1P quantification) or an Agilent 6530 quadrupole-time-of-flight MS (for analysis of Cer, SM, and DAG species). A solvent system consisting of water (eluent A) and acetonitrile/methanol (1:1, *v*/*v,* eluent B), both acidified with 0.1% formic acid, was applied for separation of sphingolipid species. Water supplemented with 1.5 mM ammonium formate and 0.1% formic acid (eluent A) and 2 mM ammonium formate and 0.15% formic acid added to methanol (eluent B) were used as mobile phase system for DAG separation. An Eclipse Plus C_8_ column (3.5 μm, 2.1 × 150 mm) guarded by a pre-column of the same material (both Agilent Technologies) was used for all measurements. Ionization occurred in electrospray ion sources operating in the positive ion mode (ESI+). The following selected reaction monitoring (SRM) transitions were used for quantification of Sph and S1P: *m*/*z* 300.3→282.3 (Sph), *m*/*z* 380.3→264.3 (S1P), *m*/*z* 307.3→289.3 (Sph-d_7_), and *m*/*z* 387.3→271.3 (S1P-d_7_). The precursor ions of Cer or SM species (differing in the lengths of their fatty acid chains) were cleaved into the fragment ions *m*/*z* 264.270 (for Cer) or *m*/*z* 184.074 (for SM), respectively [42]. Saturated DAG generated [M-H_2_O+H]^+^ ions, while unsaturated DAG preferentially formed [M+NH_4_]^+^ precursor ions: *m*/*z* 612.556 (16:0/18:1 DAG) and *m*/*z* 662.572 (18:0/20:4 DAG). Collision-induced dissociation of a fatty acid residue from the glycerol backbone yielded product ions used for specific MS/MS detection of DAG species: *m*/*z* 313.274 and *m*/*z* 339.290 (16:0/18:1 DAG), *m*/*z* 341.306 and *m*/*z* 361.274 (18:0/20:4 DAG). Quantification of cerebral lipids was performed using external calibration using the MassHunter software (Agilent Technologies). Determined lipid amounts were normalized to the actual protein content (determined via Bradford assay) of the homogenate used for extraction.

### 2.6. Mouse Neuronal Ca^2+^ Measurements

For Ca^2+^ measurements, cells were washed with HBSS+/+ (GIBCO #14025-050) and stained with 1 µM Fura-2-AM (Thermo Fisher Scientific #F1201) at 37 °C for 30 min. Afterward, cells were washed with HBSS+/+ and left for another 30 min at RT in HBSS+/+. Cover slips were taken out from 12-well plates and placed into an imaging chamber made of plexi glass. Cells were covered with HBSS+/+, and fixed to a platform adjusted at an Olympus BX51W1 immersion microscope (Olympus; Shinjuku, Tokyo, Japan) (light source: Tilluxe PX45 Xenon-light). To induce TRPC6-mediated Ca^2+^ influx into the cell soma, hyperforin 10 µM was applied. Ca^2+^ influx was recorded and visualized in TillVision Live Acquisition and Offline Analysis software [formerly FEI Munich GmbH (Till Photonics), now Thermo Fisher Scientific] as a ratio of 340/380 nm with a 40 X objective [40x/0.80 W ∞/0/FN26.5 objective (LUMPlan FL N), Olympus]. Ca^2+^-bound Fura-2 is excitable at 340 nm and the unbound state of Fura-2 at 380 nm. The ratio was calculated by analyses of emission, which was detectable at 510 nm after excitation with each wavelength. The amplitude between baseline and first plateau (ca. after 40 s past hyperforin application) was calculated and interpreted as maximum Ca^2+^ influx inducible with hyperforin 10 µM.

### 2.7. Mouse Synaptosomal Preparations and Synaptosomal Ca^2+^ Measurements

Murine ASM WT and ASM KO synaptosomes were prepared from the whole brain without the cerebellum of 10 weeks old mice as described previously [43]. Briefly, the tissues were homogenized in 15 mL ice-cold sucrose solution (0.32 M). The nuclear fraction was eliminated by centrifugation (10 min at 750× *g*, 0–4 °C), and the supernatant was centrifuged (20 min at 17,400 g; 0–4 °C) to obtain the crude synaptosomal pellets. It was then suspended in ice-cold HEPES- buffer (NaCl: 150; HEPES: 10; KCl: 6.2; Na&IPOd: 1.2; MgSO4: 1.2; glucose: 10 mM. pH 7.4 at 37 °C) aliquoted in glass tubes. Synaptosomal samples were loaded with 5 µM Fura-2-AM for 45 min. An SLM Aminco Bowman Series 2 spectrophotometer was used for measurements. For the determination of R_max_ and R_min_ 0.2% (*w*/*v*) SDS, 30 mM TRIS and 6 mM EGTA were added. Fura-2-signals were calibrated, according as earlier described using a K_D_ value of 224 nM [44].

### 2.8. Rat Cortical Phospho-CREB Immunocytochemistry

Rat cortical cells were treated either with MQ or ARC39 10 µM (ARC39 was solved in MQ) concomitantly with DMSO or hyperforin 1 µM (hyperforin was solved in DMSO) 24 h prior to fixation. ARC39 is a synthetic ASM inhibitor, which directly inhibits ASM’s catalytic activity [45,46].

Cells on cover slips were fixed with 4% (*w*/*v*) paraformaldehyde for 3 min and afterwards blocked (10% (*v*/*v*) FCS, 0.1% (*w*/*v*) glycine in PBS 1x) for 45 min. Primary antibody solution was prepared in staining solution (3% (*v*/*v*) FCS in PBS 1x): MAP 2 ms (Sigma #M4403, 1:1000), GAD 2/65 gp (Synaptic Systems #198104, 1:500; Synaptic Systems GmbH, Göttingen, Germany), pCREB rb (Ser133) (CST #9198, 1:1000; Cell Signaling Technology, Danvers, MA, USA). MAP 2 was used to stain exclusively neuronal cells. GAD 2/65 was used to distinguish between excitatory and inhibitory neurons, only cells which were negative for GAD 2/65 were taken into consideration to analyze exclusively excitatory neurons. Cover slips were incubated with primary antibody overnight at 4 °C. The next day, cover slips were washed with PBS 1x. Secondary antibody solution was prepared in staining solution: anti-mouse donkey Cy5 (Jackson ImmunoResearch #715-175-150, 1:1000; Jackson ImmunoResearch Europe Ltd., Ely, UK), anti-guinea pig donkey alexa 488 (Jackson ImmunoResearch #706-545-148, 1:1000), anti-rabbit donkey Cy3 (Jackson ImmunoResearch #711-165-152, 1:1000). Cover slips were incubated with secondary antibody for 1 h at RT. Cells were washed with PBS 1x and with MQ and finally mounted on glass slides with Fluoroshield mounting media, including DAPI (Sigma #F6057). Imaging was performed at a Nikon Eclipse Ti microscope (Nikon Corporation, Chiyoda, Tokyo, Japan). A LED Hub (Omicron-laserage Laserprodukte GmbH, Rodgau, Germany) was used as a light source, and pictures were taken as 16-bit images with an ANDOR camera (Model No: DU-885K-CSO-#VP; Andor Technology, Belfast, UK) with a 60 X objective [60x/A/1.20 WI ∞/0.15–0.18 DIC N2 WD 1.0 objective (Plan APO VC Nikon CFI); Nikon Corporation]. Images were processed with VisiView software (Visitron Systems GmbH, Puchheim, Germany) and stored as TIF files. The mean background of respective experiments of individual channels was subtracted from all pictures in ImageJ [47]. A circular region of interest was created in ImageJ, which fit the rat nuclear size displayed as DAPI staining (Ø 11 µm). All nuclei of one region that collocated to neuronal MAP 2 staining were taken into consideration, and a mean value of average pixel intensity of pCREB staining was determined per region.

### 2.9. Mouse Cortical Phospho-CREB Immunocytochemistry

Mouse cortical cells were treated with hyperforin 1 µM or DMSO for 20 min, 1 h, 6 h or 24 h. Afterwards cells were stained and analyzed accordingly to rat cortical cells. For mouse experiments, images were taken with a 20 X objective [20x/0.75 ∞/0.17 WD 1.0 objective (Plan APO Nikon CFI); Nikon Corporation] and another circular region of interest was created in ImageJ, which fit the nuclear size of a murine nucleus displayed as DAPI staining (Ø 7.5 µm). For mouse experiments, excitatory and inhibitory neurons were taken into consideration and analyzed separately.

### 2.10. Animal Welfare Declaration

All experiments were performed according to the European Committees Council Directive 2010/63/EU, German law for animal care “TierSchG” 18.05.2006 (BGBl. I S. 1206, 1313), last updated 17.12.2018 (BGBl. I S. 2586) and the German regulation for the protection of animals used for scientific purposes “TierSchVersV” 01.08.2013 (BGBl. I S. 3125, 3126), last updated 31.08.2015 (BGBl. I S. 1474).

### 2.11. Statistical Analyses

Statistical analyses were performed using GraphPad Prism 7 or GraphPad Prism 8 (GraphPad Software, LaJolla, CA, USA). Data are shown as mean ± standard error of the means (SEM). Data distribution was checked with D’Agostino Pearson omnibus normality test. Unpaired Student’s *t*-test or Mann-Whitney test was used if experiments consisted of two data sets. For considering two different parameters, two-way ANOVA was used with Sidak’s multiple comparison test. *p* ≤ 0.05 was considered as statistically significant.

## 3. Results

### 3.1. ASM Activity Is Decreased in Cortices of ASM KO Mice

**Figure 1:**
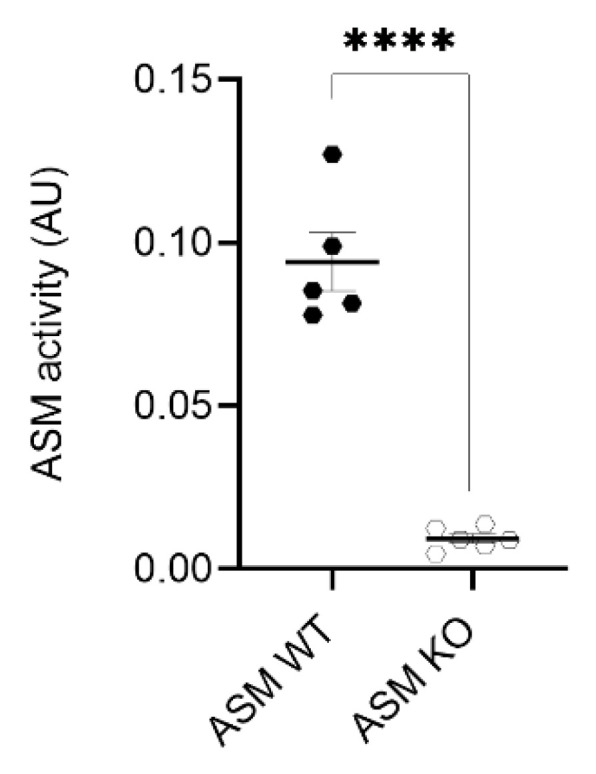
Acid sphingomyelinase (ASM) activity is decreased in cortices of ASM KO mice. ASM activity was measured in triplicates using an enzymatic activity assay. Bars indicate mean ± SEM; *n* = 5–6 animals; **** *p* ≤ 0.0001.

In the first step, we analyzed ASM activity in the cortical tissue of ASM WT and ASM KO mice. Cortices of ASM WT (*n* = 5) and ASM KO (*n* = 6) mice were isolated and subjected to an enzymatic activity assay. ASM activity in cortices of ASM KO mice was significantly decreased when compared with ASM WT (*p* < 0.0001; Student’s *t*-test; Figure 1).

### 3.2. Genetic ASM Deficiency Impacts the Sphingolipidome in the Murine Frontal Cortex

**Figure 2:**
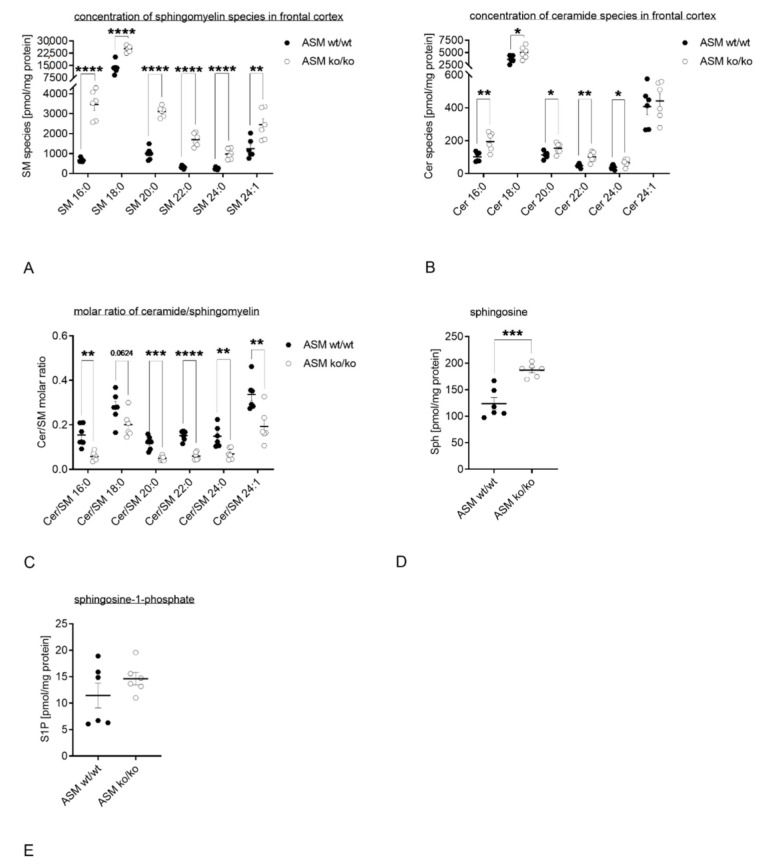
Genetic ASM deficiency impacts the sphingolipidome in the murine frontal cortex. (**A**). Concentrations of all analyzed SM species were significantly increased. (**B**). The concentrations of Cer 16:0, Cer 18:0, Cer 20:0, Cer 22:0, as well as Cer 24:0 were elevated. (**C**). The overall molar ratio of Cer/SM was decreased in ASM KO mice compared with WT mice. (**D**). The concentrations of sphingosine were significantly increased in ASM KO mice vs. ASM WT. (**E**). The concentration of S1P did not show any significant change in ASM KO mice. Bars indicate mean ± SEM; *n* = 6 animals; * *p* ≤ 0.05, ** *p* ≤ 0.01, *** *p* ≤ 0.001, **** *p* ≤ 0.0001.

For this study, we used an ASM KO mouse model and analyzed, if ASM deficiency resulted in changes in the sphingolipidome. Frontal cortices of ASM WT and ASM KO mice, were isolated and analyzed using mass spectrometry. Several species of specific sphingolipids were analyzed: sphingomyelin (SM) 16:0, SM 18:0, SM 20:0, SM 22:0, SM 24:0, SM 24:1, ceramide (Cer) 16:0, Cer 18:0, Cer 20:0, Cer 22:0, Cer 24:0, Cer 24:1, sphingosine (Sph), sphingosine1-phosphate (S1P). In the frontal cortices of ASM KO mice compared with ASM WT mice, concentrations of SM 16:0, SM 18:0, SM 20:0, SM 22:0, SM 24:0 and SM 24:1 were elevated (*p*_SM 16:0_ < 0.0001, *p*_SM 18:0_ < 0.0001, *p*_SM 20:0_ < 0.0001, *p*_SM 22:0_ < 0.0001, *p*_SM 24:0_ < 0.0001, *p*_SM 24:1_ = 0.0080; Student’s *t*-test; Figure 2A). Moreover, the concentrations of Cer 16:0, Cer 18:0, Cer 20:0, Cer 22:0 and Cer 24:0 were increased in ASM KO mice vs. ASM WT mice (*p*_Cer 16:0_ = 0.0045, *p*_Cer 18:0_ = 0.0371, *p*_Cer 20:0_ = 0.0256, *p*_Cer 22:0_ = 0.0021, *p*_Cer 24:0_ = 0.0230, *p*_Cer 24:1_ = 0.6269; Student’s *t*-test; Figure 2B). When looking at the ratio between ceramide and SM, ASM KO mice displayed a significant decreased ratio compared with ASM WT mice for Cer/SM 16:0, Cer/SM 20:0, Cer/SM 22:0, Cer/SM 24:0 and Cer/SM 24:1 in their frontal cortices (*p*_Cer/SM 16:0_ = 0.0014, *p*_Cer/SM 18:0_ = 0.0624, *p*_Cer/SM 20:0_ = 0.0004, *p*_Cer/SM 22:0_ < 0.0001, *p*_Cer/SM 24:0_ = 0.0048, *p*_Cer/SM 24:1_ = 0.0072; Student’s *t*-test; Figure 2C). Concentration of sphingosine was elevated in ASM KO mice vs. ASM WT mice (*p*_Sph_ = 0.0005; Student’s *t*-test; Figure 2D). Deletion of ASM showed no significant impact on S1P concentrations (*p*_S1P_ = 0.2561; Student’s *t*-test; Figure 2E).

**Figure 3:**
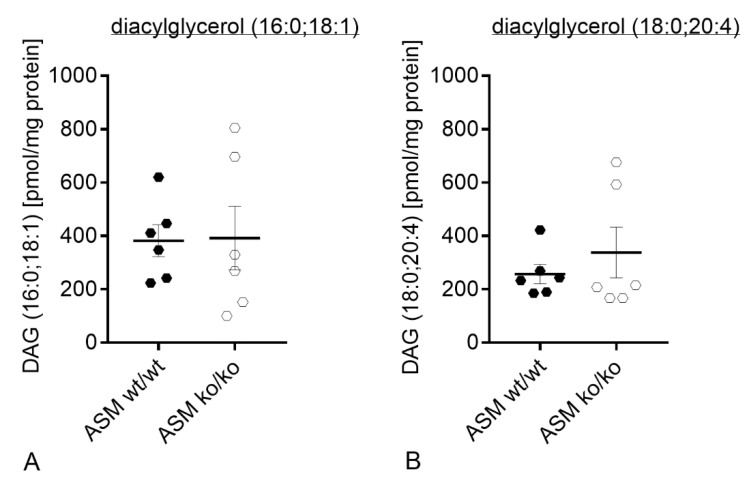
Genetic ASM deficiency does not affect diacylglycerol levels in the murine frontal cortex. (**A**). Deletion of ASM showed no significant impact on DAG (16:0; 18:1) or (**B**). DAG (18:0; 20:4) concentrations in frontal cortex. Bars indicate mean ± SEM; *n* = 6 animals.

During sphingomyelin biosynthesis diacylglycerols (DAG) are generated that are endogenous TRPC6 activators [48]. Therefore, the concentrations of DAG 16:0; 18:1 (Figure 3A) and DAG 18:0; 20:4 (Figure 3B) in frontal cortices of ASM KO mice compared with ASM WT mice were analyzed. ASM deficiency did not introduce any changes in both DAG species (*p*_DAG (16:0; 18:1)_ = 0.9408, *p*_DAG (18:0; 20:4)_ = 0.4433; Student’s *t*-test).

### 3.3. Genetic ASM Deficiency Decreases Hyperforin-Induced Ca2^+^ Influx in Primary Murine Neuronal Cells and Synaptosomes

**Figure 4:**
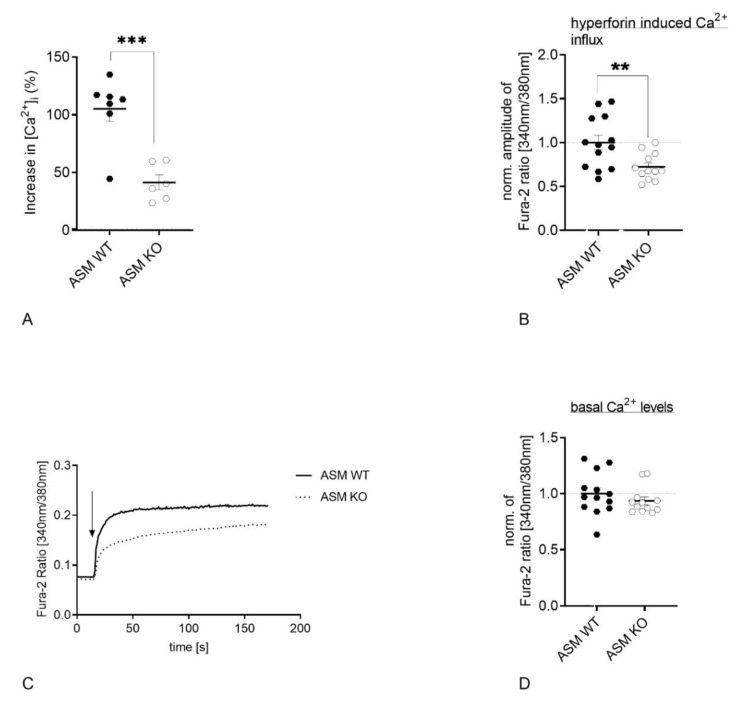
Genetic ASM deficiency decreases TRPC6-mediated Ca^2+^ influx in synaptosomes and cultured primary murine neuronal cells. (**A**). Hyperforin-induced (10 µM) Ca^2+^ influx in murine brain synaptosomes, isolated from ASM KO mice, was reduced compared with synaptosomes isolated from ASM WT mice (*n* = 6 animals). (**B**). Application of hyperforin 10 µM activates TRPC6-mediated Ca^2+^ influx. The amplitude of change in intracellular Ca^2+^ concentration is diminished in cortical ASM KO neurons compared with cortical ASM WT. (**C**). Representative time-curve of TRPC6-mediated Ca^2+^ influx in cortical mouse neurons. Arrow indicates the application of hyperforin 10 µM. (**D**). Baseline Ca^2+^ concentrations of ASM KO and ASM WT cortical neurons do not differ. Bars indicate mean ± SEM; *n* = 4 cell batches, 1–4 cover slips per batch were analyzed; ** *p* ≤ 0.01, *** *p* ≤ 0.001.

Under physiological conditions, exogenously applied hyperforin activates TRPC6 and induces an immediate cation entry into the cell soma [30]. Loading of cells with Fura-2 and subsequent application of hyperforin (10 µM) detects the Ca^2+^ influx using fluorescence microscopy technique. Synaptosomes, which were isolated from ASM KO mice were compared to synaptosomes isolated from ASM WT mice. ASM deficiency introduced a significant decrease in hyperforin-activated Ca^2+^ influx (*p* = 0.0005; Student’s *t*-test; Figure 4A). A similar pattern could be observed during measurements of internal Ca^2+^ concentrations of cultured cortical neurons. Neurons of ASM-deficient mice revealed a decrease in TRPC6-mediated Ca^2+^ influx compared to cortical neurons derived from ASM WT animals (*p* = 0.0081; Student’s *t*-test; Figure 4B,C), while baseline levels of Ca^2+^ were not changed (*p* = 0.1683; Mann-Whitney test; Figure 4D).

**Figure 5:**
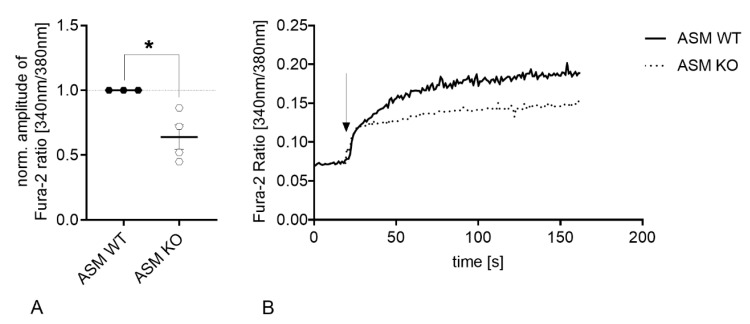
Co-culturing of ASM WT glial cells with ASM KO neurons does not rescue TRPC6 phenotype. (**A**). Ca^2+^ influx is reduced in ASM-deficient cortical mouse neurons, which were grown together with ASM WT glial cells compared with ASM WT neurons, which were grown with ASM WT glial cells in a Banker’s culture. (**B**). Representative time-curve of TRPC6-mediated Ca^2+^ influx in cortical mouse neurons, which were grown in a Banker’s culture with ASM WT glial cells. Arrow indicates the application of hyperforin 10 µM. Bars indicate mean ± SEM; *n* = 3 cell batches, 1–2 cover slips per batch were analyzed; * *p* ≤ 0.05.

To investigate if the diminished Ca^2+^ influx in ASM KO neurons could be rescued by the presence of ASM WT glia cells, neurons of both genotypes were plated in a Banker’s culture concomitantly with ASM WT glial cells. However, the TRPC6-mediated Ca^2+^ influx in ASM KO neurons was reduced compared with ASM WT neurons, which indicates that the presence of ASM WT glial cells was not sufficient to rescue the Ca^2+^ flux phenotype of ASM KO neurons (*p* = 0.0234; Student’s *t*-test; Figure 5).

### 3.4. ASM Activity Inhibition Prevents Hyperforin-Induced CREB Phosphorylation in Primary Rat Neuronal Cells

**Figure 6:**
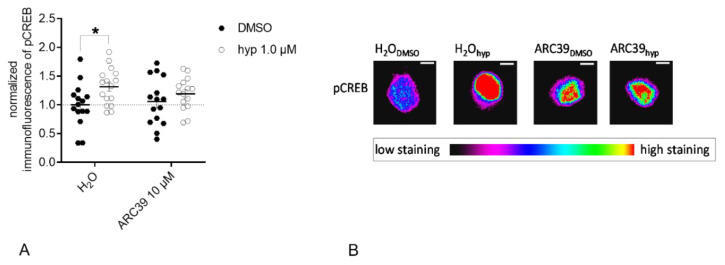
ASM activity inhibition prevents hyperforin-induced CREB phosphorylation in primary rat neuronal cells. (**A**). Hyperforin (1 µM) induced CREB phosphorylation in rat excitatory cortical neurons after 24 h of treatment. This effect was partly abolished when ASM activity was inhibited with ARC39 10 µM (**B**). Representative images of pCREB staining in single nuclei of excitatory rat neurons after indicated treatments. Scale bar indicates 5 µm. Bars indicate mean ± SEM; *n* = 1 cell batch, 3 cover slips analyzed per batch, 5 regions analyzed per cover slip; * *p* ≤ 0.05.

Observed differences in Ca^2+^ influx led to the question if modulation of TRPC6-mediated Ca^2+^ influx by ASM might influence CREB phosphorylation downstream to TRPC6 activation. To assess the impact of ASM on phosphorylation of CREB downstream of TRPC6, we used rat primary cortical neurons as a first piloting experiment. First, we investigated if pharmacological ASM activity inhibition affects the TRPC6-induced changes in CREB phosphorylation of excitatory neurons. ASM activity was inhibited with ARC39 10 µM before application of TRPC6 agonist hyperforin 1 µM or vehicle (DMSO). The nuclear pCREB was assessed 24 h later. Hyperforin treatment induced CREB phosphorylation in rat excitatory cortical neurons after 24 h of treatment (*p*_H20: DMSO vs. hyp_ = 0.0294, Sidak’s test). The treatment with ARC39 significantly attenuated the hyperforin-induced nuclear pCREB (*p*_ARC39: DMSO vs. hyp_ = 0.5137, Sidak’s test; two-way ANOVA: interaction: F(1.57) = 1.043, *p* = 0.3114; ASM inhibition: F(1.57) = 0.1383, *p* = 0.7114; treatment: F(1.57) = 6.27, *p* = 0.0152); Figure 6A). Representative images of pCREB staining in single nuclei of rat neurons after indicated treatments can be found in Figure 6B. Similar results were obtained when ARC39 was applied for 48 h (data not shown).

### 3.5. Genetic ASM Deficiency Impacts Hyperforin-Induced CREB-Phosphorylation in Primary Murine Neuronal Cells

**Figure 7:**
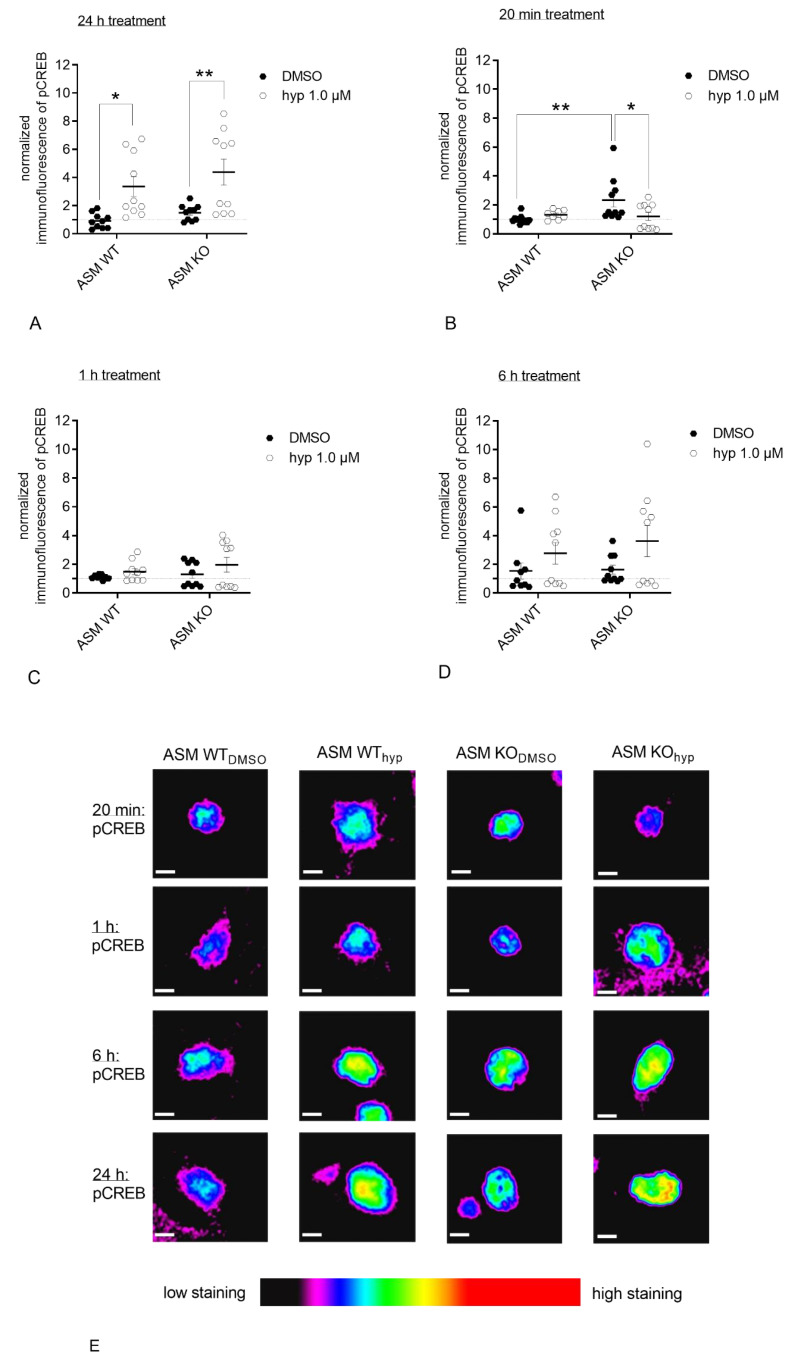
Genetic ASM deficiency impacts hyperforin-induced CREB-phosphorylation in excitatory primary murine neuronal cells. (**A**). 24 h after hyperforin (1 µM) stimulation, both ASM WT and ASM KO excitatory neurons showed a significant increase in pCREB staining compared with control conditions. (**B**). After a 20 min treatment of ASM KO and ASM WT neurons with hyperforin, distinctive hyperforin-induced changes were visible. While in ASM WT neurons after hyperforin application, a small positive trend was visible towards stronger pCREB staining, ASM KO neurons contrarily showed significantly decreased pCREB levels. (**C**). 1 h after hyperforin application, no treatment or genotype-induced effects were observed. (**D**). 6 h after hyperforin stimulation, no treatment or genotype-induced effects were observed. (**E**) Representative images of pCREB staining in single nuclei of excitatory murine neurons after indicated treatments and time points. Scale bar indicates 5 µm. Bars indicate mean ± SEM; *n* = 2 cell batches, 1 cover slip analyzed per batch, 3–7 regions analyzed per cover slip; * *p* ≤ 0.05, ** *p* ≤ 0.01.

In a next experiment, we assessed the effect of chronical ASM activity removal upon deletion of the *Smpd1* gene. To this end, we prepared primary cortical neurons from ASM KO mice and their WT siblings. Dynamic aspects of CREB phosphorylation after hyperforin 1 µM application were investigated in excitatory and in inhibitory neurons. Four specific time points of hyperforin stimulation (20 min, 1 h, 6 h, 24 h) were analyzed. When stimulated with hyperforin, similar to the results obtained in rat neurons after 24 h of hyperforin application, ASM WT excitatory neurons showed a significant increase in pCREB staining. Moreover, ASM KO excitatory neurons showed this effect, in contrast to the results in rat excitatory neurons (*p*_ASM WT; DMSO vs. hyp_ = 0.0118; *p*_ASM KO; DMSO vs. hyp_ = 0.0029, Sidak’s test; Figure 7A). Two-way ANOVA revealed an effect for treatment, but no effect for interaction or genotype [interaction: F(1.36) = 0.135, *p* = 0.7154; genotype: F(1.36) = 1.82, *p* = 0.1857; treatment: F(1.36) = 20.31, *p* < 0.0001]. Since CREB phosphorylation is a quick response, we analyzed CREB phosphorylation after 20 min of hyperforin application to account for the physiologic time frame. After 20 min of hyperforin application, ASM WT excitatory neurons showed a trend towards increased pCREB staining compared with control conditions. ASM KO excitatory neurons showed a significant decrease in pCREB staining (*p*_ASM KO: DMSO vs. hyp_ = 0.0175, Sidak’s test; Figure 7B). Two-way ANOVA revealed an effect for genotype-treatment interaction and genotype, and no effect for treatment [interaction: F(1.36) = 6.143, *p* = 0.0180; genotype: F(1.36) = 4.414, *p* = 0.0427; treatment: F(1.36) = 1.961, *p* = 0.1700]. After 1 h (Figure 7C) and 6 h (Figure 7D) of hyperforin stimulation, no significant effects of treatment or genotype were observed (1 h: Two-way ANOVA revealed no effect for interaction, genotype or treatment [interaction: F(1.35) = 0.2107, *p* = 0.6490; genotype: F(1.35) = 1.073, *p* = 0.3074; treatment: F(1.35) = 2.616, *p* = 0.1148]; 6 h: Two-way ANOVA revealed an effect for treatment, but no effect for interaction or genotype [interaction: F(1.35) = 0.2636, *p* = 0.6109; genotype: F(1.35) = 0.3976, *p* = 0.5324; treatment: F(1.35) = 4.688, *p* = 0.0373]). Representative images of pCREB staining in single nuclei of excitatory murine neurons after indicated treatments and time points can be seen in Figure 7E.

**Figure 8:**
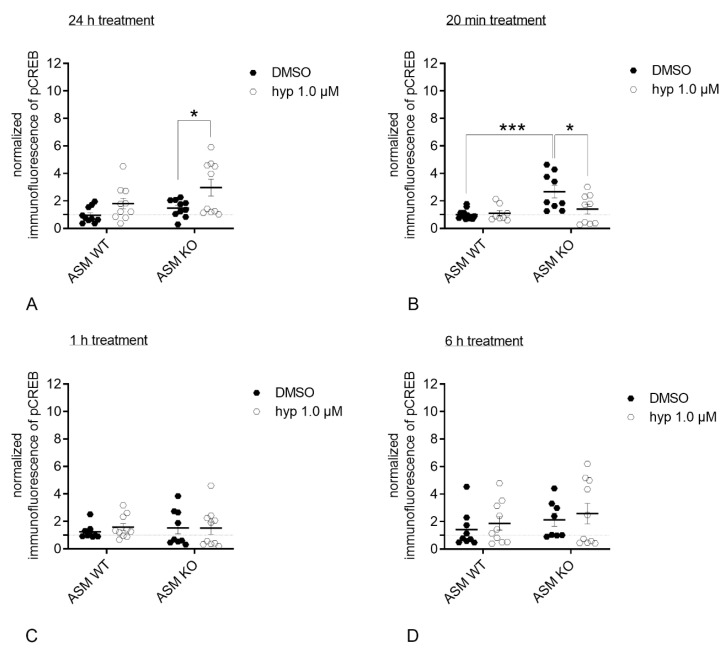

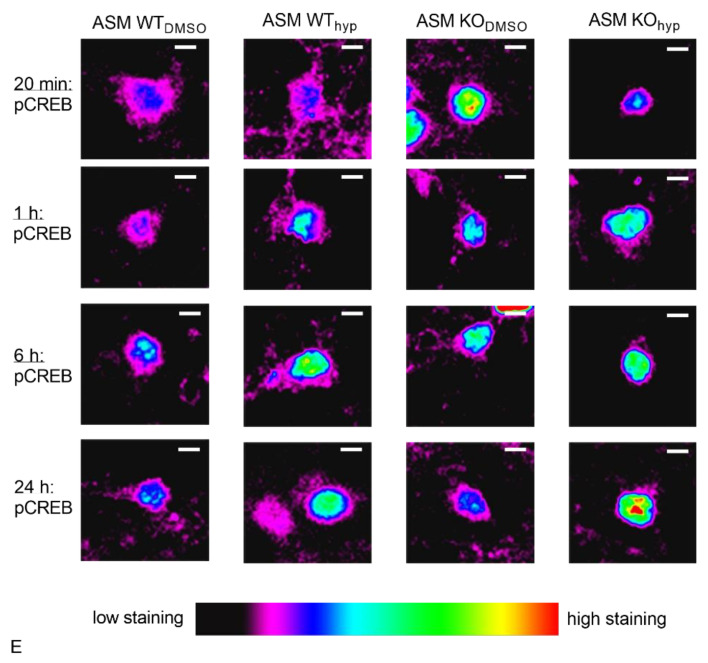
Genetic ASM deficiency impacts hyperforin-induced CREB-phosphorylation in inhibitory primary murine neuronal cells. (**A**). 24 h after hyperforin (1 µM) stimulation, ASM KO inhibitory neurons showed a significant increase in pCREB staining, ASM WT neurons a trend towards an increase. (**B**). After a 20 min treatment, ASM WT neurons showed a small positive trend towards stronger pCREB staining, and ASM KO neurons contrarily showed significantly decreased pCREB levels. (**C**). 1 h after hyperforin application, no treatment or genotype-induced effects were observed. (**D**). 6 h after hyperforin stimulation, no treatment or genotype-induced effects were observed. (**E**). Representative images of pCREB staining in single nuclei of excitatory murine neurons after indicated treatments and time points. Scale bar indicates 5 µm. Bars indicate mean ± SEM; *n* = 2 cell batches, 1 cover slip analyzed per batch, 3–7 regions analyzed per cover slip; * *p* ≤ 0.05, *** *p* ≤ 0.001.

Similar results were obtained in murine inhibitory neuronal cells. After 24 h of hyperforin 1 µM treatment, ASM WT inhibitory neurons exhibited a non-significant positive trend, while in ASM KO cells, there was a significant increase in pCREB levels compared with control conditions (*p*_ASM WT: DMSO vs. hyp_ = 0.2405; *p*_ASM KO: DMSO vs. hyp_ = 0.0191, Sidak’s test; Figure 8A). Two-way ANOVA revealed an effect for genotype and treatment, but no effect for interaction [interaction: F(1.36) = 0.6968, *p* = 0.4094; genotype: F(1.36) = 4.753, *p* = 0.0359; treatment: F(1.36) = 9.211, *p* = 0.0045]. The 20 min treatment with hyperforin 1 µM resulted in a trend towards increased pCREB levels in ASM WT cells, and decreased CREB phosphorylation in ASM-deficient cells (*p*_ASM KO: DMSO vs. hyp_ = 0.0103, Sidak’s test; Figure 8B). Two-way ANOVA revealed an effect for interaction and genotype, and no effect for treatment [interaction: F(1.34) = 5.32, *p* = 0.0273; genotype: F(1.34) = 11.31, *p* = 0.0019; treatment: F(1.34) = 3.949, *p* = 0.0550]. After 1 h (Figure 8C) and 6 h (Figure 8D) of hyperforin treatment, no significant differences were observed regarding treatment and genotypes (1 h: Two-way ANOVA revealed no effect for interaction, genotype or treatment [interaction: F(1.34) = 0.2579, *p* = 0.6149; genotype: F(1.34) = 0.0834, *p* = 0.7745; treatment: F(1.34) = 2217, *p* = 0.6408]; 6 h: Two-way ANOVA revealed no effect for interaction, genotype or treatment [interaction: F(1.33) = 0.00005, *p* = 0.9943; genotype: F(1.33) = 1.577, *p* = 0.2180; treatment: F(1.33) = 0.6351, *p* = 0.4312]). Representative images of pCREB staining in single nuclei of inhibitory murine neurons after indicated treatments and time points are shown in Figure 8E.

## 4. Discussion

In our study, sphingolipidomic analyses of frontal cortices of ASM KO mice revealed a complex dysregulation of the ceramide/sphingomyelin system, including a decreased Cer/SM molar ratio. The Ca^2+^ influx through TRPC6 induced by an application of its agonist hyperforin was impaired in ASM KO cortical neurons and brain synaptosomes in Ca^2+^ imaging experiments. Analogous, TRPC6 downstream signaling induced by 20 min of hyperforin application was impaired in ASM KO cortical excitatory and inhibitory neurons as indicated by decreased levels of immunocytochemistry staining of CREB phosphorylation in nuclei. The potential mediator between ASM and TRPC6 systems, DAG, however, was not affected by ASM knockout in frontal murine cortices.

To approach the effect of ASM knockout on lipid levels in the cortical brain region, we analyzed several sphingolipids. As expected, ceramide to SM ratios were decreased in ASM KO cortices (Figure 2C). Of note, ceramide levels were increased in ASM KO mice, but to a lower extent than sphingomyelin levels (Figure 2A,B). This points to a complex counter regulation of sphingolipid metabolism induced by other enzymes of the rheostat after the deletion of one of the metabolizing enzymes. Moreover, sphingosine levels were increased in ASM KO cortices, which might be a result of counter regulations (Figure 2D). Increased levels of SM in ASM KO brain tissue have been already described by other studies, with a focus on non-lysosomal membranes [49]. Here, the main SM species was SM 18:0 [50]. SM 18:0 was earlier found to be involved in a specific lipid-protein transmembrane domain interaction [51]. This points to a specific role for each lipid species, which has just been started to be under investigation [52]. Other studies investigated gangliosides in brain lysates of ASM KO mice and found GM2 and GM3 levels to be increased [53,54]. Therefore, the deletion of the *Smpd1* gene, resulting in ASM deficiency, results in sphingolipid dysregulation in brain tissues and might be responsible for severe changes in membrane composition and membrane fluidity. Importantly, ASM deficiency not only affects lysosomal catabolism, but also plasma membrane integrity, which has an impact on the function of membrane proteins. In MDD, high ASM/ceramide levels might negatively regulate TRPC6 activity, which could be balanced by antidepressant-mediated ASM inhibition via restoration of physiological sphingolipid composition of the membrane.

Ion currents depend on the optimal function of membrane proteins, for example, TRPC6 channels. Ca^2+^ currents are of the highest importance for the communication between neurons. Accordingly, impairment of Ca^2+^ currents is associated with neuronal malfunctions. Thus, we investigated murine cortical neurons, which exhibited no residual ASM activity. In ASM KO neurons, hyperforin-induced Ca^2+^ influx was significantly reduced compared with ASM WT cortical neurons (Figure 4B). Indeed, we were able to show for the first time that ASM dysregulation impacts TRPC6 function in neurons. Thus, both systems seem to be interconnected. Our earlier study in a neuronal cell line included data on FIASMA-mediated impaired TRPC6 function that was rescued by the application of bacterial sphingomyelinase [33]. Therefore, we thought ASM WT glial cells would be able to rescue the impaired TRPC6 function in ASM KO neurons with the secretion of ASM from WT glia to KO neurons. However, WT glial cells could not recover TRPC6 function (Figure 5A). Either ASM secretion from glia cells was not sufficient, or the complete absence of ASM in the KO model has a too strong phenotype, in contrast to a 50% decrease in ASM activity achieved by FIASMA application.

To assess potential downstream effects of dysregulated TRPC6 function that could impact neuronal integrity, we analyzed CREB phosphorylation as an important target of TRPC6 activity. TRPC6-mediated Ca^2+^ influx results in the activation of different kinase pathways, which converge in the activation of the transcriptional factor CREB [29]. CREB, in turn, induces the transcription of genes that are relevant for neuronal processes, among them BDNF signaling [55]. Hyperforin was earlier shown to induce CREB phosphorylation [29,56]. In our experiment analyzing excitatory primary rat neurons, the stimulation with hyperforin resulted in a significant increase in pCREB after 24 h (Figure 6A). Modulation of ASM activity using a direct pharmacological inhibitor abolished the induction of pCREB (Figure 6A). To validate this effect under fully ASM deficient conditions, we cultured ASM KO cortical mouse neurons, stimulated cells with hyperforin, and analyzed pCREB staining of excitatory and inhibitory neurons. Results were dependent on the duration of hyperforin stimulation. Whereas, after 24 h of hyperforin stimulation pCREB was induced in ASM WT neurons as described for rat neurons, the effect of ASM inhibition was different (Figure 7A). The ASM knockout resulted in an increase in pCREB after 24 h of hyperforin stimulation (Figure 7A), whereas the pharmacological inhibition of ASM in rat neurons prevented the increase in pCREB as seen for wildtype conditions (Figure 6A). Thus, ASM knockout effects seem to be different from temporary and short-term inhibition of ASM activity by inhibitors. However, CREB activation under physiologic conditions is a very quick process. Thus, the more physiologic time point of CREB phosphorylation seems to be after 20 min of hyperforin stimulation. In this case, we see a trend towards increased CREB phosphorylation for ASM WT neurons, and a significant decrease of pCREB in ASM KO cells induced by hyperforin (Figure 7B). Interestingly, pCREB was significantly increased in ASM KO excitatory neurons compared with ASM WT neurons already under control conditions (Figure 7B). Under ASM deficient conditions, hyperforin stimulation is not sufficient to activate Ca^2+^currents via TRPC6 and cannot activate the downstream signaling target CREB, but instead normalizes dysregulated CREB phosphorylation (Figure 7B). The described effects seem to hold true for both excitatory (Figure 7A–D) and inhibitory neurons (Figure 8A–D), even though TRPC6 has been found previously mainly at excitatory synapses [26]. An explanation for our results could be that ASM deficiency, in general, affects protein distribution to dendrites and axons. Thus, in ASM KO neurons proteins were located at compartments, where they usually do not appear under physiological conditions [49]. Analogous, TRPC6 expression and trafficking could be differentially regulated in ASM KO neurons, which might lead to observed effects in CREB phosphorylation. Several findings indicate that the phosphorylation of CREB can be regulated by the depolarization of neurons. Accordingly, different phosphorylation sites could be used to activate CREB in different time contexts [57]. The CREB phosphorylation at Ser133 can be elicited by different stimuli, while the phosphorylation at position Ser142 or Ser143 depends on Ca^2+^ currents of L-VSCCs or NMDA receptors. Cell depolarization promotes Ser142 and Ser143 phosphorylation, which further facilitates CREB activation. An immediate and short-term increase in Ser133 pCREB levels seems to be regulated by CaMK IV, while the RAS pathway induced a long-term action and also the CREB phosphorylations at Ser142 and Ser143 represent a prolonged reaction [57,58]. Under our physiological conditions, CREB phosphorylation in ASM WT and ASM KO cells mainly diverges in the short-term effects and not in the long term CREB phosphorylation. Since short term CREB phosphorylation mostly relates to CaMK IV activation, this could therefore be the major molecular effector responsible for pCREB level differences in ASM WT and ASM KO neurons. Further studies are needed to fill in other important molecular players in this pathway.

A mediator between ASM and TRPC6 effects could be DAG. It was shown that TRPC6 channels can be activated by either exogenous hyperforin or endogenous diacylglycerol. The elicited non-selective cation currents can consist of Na^+^ and Ca^2+^ ions [30]. Both ion currents are supposed to mediate the antidepressive action of hyperforin. TRPC6-mediated Na^+^ influx was shown to inhibit monoamine reuptake out of the synaptic cleft [59], while Ca^2+^ influx promotes neuronal plasticity [29]. Sphingomyelin biosynthesis via sphingomyelin synthase generates DAG [48]. Thus, we measured DAG species in murine cortices and hypothesized changes in ASM KO mice. Nevertheless, DAG levels were not affected by ASM deficiency (Figure 3A,B). Since sphingosine levels were influenced by ASM knockout (Figure 2D), this lipid could play a role in TRPC6 recruitment to lipid rafts. Further studies should investigate the role of sphingosine in TRPC6 biology.

ASM and TRPC6 are both proteins that have implications at nerve termini and synapses, and the pathology of MDD is highly related to synaptic function [60]. While ASM is claimed to induce presynaptic activity by the generation of ceramide and the subsequent facilitation of sphingosine production [14], TRPC6 has been shown to be highly involved in spine formation and also enhances miniature synaptic transmission [24]. We showed that hyperforin-mediated Ca^2+^ influx was impaired in brain synaptosomes (Figure 4A). Thus, the interaction of ASM and TRPC6 especially in the context of synaptic function could deepen our understanding of pathologies in MDD. Under ASM deficiency, a strong degenerated spine phenotype connected to impaired stability of cytoskeleton was described [61]. Moreover, a smaller size of synapses and reduced synaptic signaling was observed in ASM KO neurons [62]. This could be in line with the mental retardation that was described in Niemann-Pick disease type A patients who have a residual ASM activity of below 5% [53]. Moreover, the FIASMA antidepressant fluoxetine has been shown to beneficially modulate synaptic dynamics [63]. At the synapse, the synaptic vesicles are tightly regulated by the phosphorylation pattern of synapsin 1, which is, in turn, controlled by kinases that depend on internal Ca^2+^ concentrations [64]. Since TRPC6 regulates intracellular Ca^2+^ levels, its activation could be involved in vesicle recycling. The potential interaction of ASM and TRPC6 at the synapse might serve as a promising research topic for the future to better understand MDD pathology.

## Abbreviations

**Abbreviation**	**Full Name**
Anti/anti	antibiotic/antimycotic
ARC39	C10 bisphosphonate 1-aminodecane-1,1-bisphosphonic acid
ASM	acid sphingomyelinase
ASM KO	ASM-knockout
ASM WT	ASM-wildtype
Cer	ceramide
CREB	cAMP response element-binding protein
DAG	diacylglycerol
DAPI	4′,6-diamidino-2-phenylindole
DIV	days in vitro
DMEM	Dulbecco’s Modified Eagle’s Medium
DMSO	dimethyl sulfoxide
E 18	embryonic day 18
EGTA	ethylene glycol-bis(β-aminoethyl ether)-N,N,N′,N′-tetraacetic acid
FCS	fetal calf serum
Fura-2-AM	Fura-2-acetoxymethylester
GAD2/65	glutamic acid decarboxylase 2/65
gp	guinea pig
HBSS-/-	Hank’s balanced salt solution without calcium and magnesium
HBSS+/+	Hank’s balanced salt solution with calcium and magnesium
HPLC	high-performance liquid chromatography
hyp	hyperforin
MAP 2	microtubule associated protein 2
MDD	major depressive disorder
MQ	Millipore water
ms	mouse
MS	mass spectrometry
NB	Neurobasal
NBA	Neurobasal A
P	postnatal age
PBS	phosphate buffered saline
PC12	pheochromocytoma 12 cell line
PCR	polymerase chain reaction
pCREB	phospho-CREB (serine 133)
PPD-mix	papain-dispase-DNase mixture
rb	rabbit
RT	room temperature (20–22 °C)
S1P	sphingosine 1-phosphate
SRM	selected reaction monitoring
SDS	sodium dodecylsulfate
SM	sphingomyelin
Sph	sphingosine
TBST	TRIS buffered saline + 1% (*v*/*v*) Tween
TRIS	tris(hydroxymethyl)-aminomethan
TRPC6	canonical transient receptor potential channel 6
USA	United States of America
UV	ultraviolet light
vs.	versus
